# MP2RAGE vs. MPRAGE surface-based morphometry in focal epilepsy

**DOI:** 10.1371/journal.pone.0296843

**Published:** 2024-02-08

**Authors:** Cornelius Kronlage, Ev-Christin Heide, Gisela E. Hagberg, Benjamin Bender, Klaus Scheffler, Pascal Martin, Niels Focke

**Affiliations:** 1 Department of Neurology and Epileptology, Hertie Institute for Clinical Brain Research, University of Tuebingen, Tuebingen, Germany; 2 Clinic of Neurology, University Medical Center Goettingen, Goettingen, Germany; 3 High-Field MR Centre, Max-Planck-Institute for Biological Cybernetics, Tuebingen, Germany; 4 Department for Biomedical Magnetic Resonances, University of Tuebingen, Tuebingen, Germany; 5 Department of Neuroradiology, University of Tuebingen, Tuebingen, Germany; Jikei University School of Medicine, JAPAN

## Abstract

In drug-resistant focal epilepsy, detecting epileptogenic lesions using MRI poses a critical diagnostic challenge. Here, we assessed the utility of MP2RAGE–a T1-weighted sequence with self-bias correcting properties commonly utilized in ultra-high field MRI–for the detection of epileptogenic lesions using a surface-based morphometry pipeline based on FreeSurfer, and compared it to the common approach using T1w MPRAGE, both at 3T. We included data from 32 patients with focal epilepsy (5 MRI-positive, 27 MRI-negative with lobar seizure onset hypotheses) and 94 healthy controls from two epilepsy centres. Surface-based morphological measures and intensities were extracted and evaluated in univariate GLM analyses as well as multivariate unsupervised ‘novelty detection’ machine learning procedures. The resulting prediction maps were analyzed over a range of possible thresholds using alternative free-response receiver operating characteristic (AFROC) methodology with respect to the concordance with predefined lesion labels or hypotheses on epileptogenic zone location. We found that MP2RAGE performs at least comparable to MPRAGE and that especially analysis of MP2RAGE image intensities may provide additional diagnostic information. Secondly, we demonstrate that unsupervised novelty-detection machine learning approaches may be useful for the detection of epileptogenic lesions (maximum AFROC AUC 0.58) when there is only a limited lesional training set available. Third, we propose a statistical method of assessing lesion localization performance in MRI-negative patients with lobar hypotheses of the epileptogenic zone based on simulation of a random guessing process as null hypothesis. Based on our findings, it appears worthwhile to study similar surface-based morphometry approaches in ultra-high field MRI (≥ 7 T).

## 1 Introduction

In drug-resistant focal epilepsy, epilepsy surgery represents a treatment option that can achieve seizure freedom in a significant percentage of eligible patients [1]. The identification of the epileptogenic zone (EZ) poses a crucial diagnostic challenge. Besides non-invasive video-EEG monitoring and neuropsychological assessment, magnetic resonance imaging (MRI) is a cornerstone of the presurgical workup and allows identification of focal structural abnormalities such as focal cortical dysplasia (FCD). The presence of distinct, visible lesions (i.e., MRI-positive cases) is known to be associated with better surgical outcome [2]. In MRI-negative patients, a hypothesis on the localization of the EZ can often only be formulated on the lobar level. Even with further elaborate investigations such as PET, ictal SPECT, high-density-EEG, MEG and invasive intracranial EEG, surgery is less likely to be performed and outcomes are less favourable [3].

Advances are being made toward improving the detection of epileptogenic lesions on MRI both in terms of image acquisition and analysis. Concerning the former, for instance, novel contrasts and measurements at ultra-high field strengths (7T) have been proposed for delineating epileptogenic lesions [4]. At ultra-high field, conventional T1-weigthed sequences like MPRAGE are compromised by inhomogeneity artefacts. Hence, MP2RAGE (Magnetization-Prepared 2 Rapid Gradient-Echo) is frequently employed since this specialized acquisition sequence generates T1-weighted images which are ‘self bias-field corrected’ [5, 6]. Besides its use at ultra-high field, recent studies suggest that this sequence has improved lesion detection in focal epilepsy also at clinical field strengths of 3T [7–10].

In addition to visual reading, various image processing methods for (automated) detection of epileptogenic lesions have been devised. (It should be noted, however, that a direct comparison of detection performance is impeded by software not being openly shared in many cases, as well as the lack of a publicly available benchmark dataset.) Initially, pipelines used univariate statistical procedures based on voxel-based morphometry, and more recently, and surface-based morphometry. In the last years, machine learning approaches have been applied to this task [4]: In most works, a number of hand-crafted FCD sensitive features are obtained by established segmentation pipelines per-voxel or per-vertex and classifiers like shallow neural networks are trained on manually segmented lesional and healthy voxels/vertices to allow prediction of lesions in unseen subjects [11–13]. In large multicentre datasets, two such approaches have been reported to yield sensitivity/specificity values of 81.0/84.3% [11] and 59/54% [12]. In such techniques, information about the neighbourhood of each voxel/vertex is only considered indirectly through spatial smoothing operations in pre-processing; a graph convolutional neural network–with the ability to explicitly model spatial relationships–operating on surface features has been shown to improve lesion detection performance [14]. Skipping manual feature extraction, deep convolutional neural networks (processing patches of voxel data directly) have also been applied to the task and shown promising performance [15].

For epileptogenic lesion detection, in contrast to supervised machine learning, where training is usually performed with lesional FCD cases, it is also conceivable to apply unsupervised techniques that estimate the probability that a case lies outside the distribution of healthy controls. Multivariate statistical techniques are possible: Non-parametric combination (NPC) has recently been applied to detecting epileptogenic lesions using voxel-based features [16] (although it remains unclear to us how to achieve a sufficient number of permutations when comparing one subject to a control group). Similarly, a one-class SVM (support vector machine) can be used to classify voxel-based features [17]. This method has been extended by a deep convolutional representation learning technique for patch- or voxel-wise feature extraction [18]. We are not aware of any unsupervised learning approaches using surface-based features for epileptogenic lesion identification so far.

Automated detection is most clinically relevant in visually MRI-negative patients. However, in these patients, defining the ground truth for evaluating detection results is difficult. The most insightful cases are those without any visible finding but with positive post-operative histology. Yet, this constellation is not very frequent and in many instances, MRI-negativity is defined as ‘upon initial routine radiological assessment’ [19] or ‘ever reported MRI-negative’ [12], so that some of these lesions may be subtle but still visible on MRI. An alternative approach, as applied previously [9, 20], is to evaluate the concordance of detection results with hypotheses concerning the EZ based on available results of the presurgical diagnostic workup, even if no surgery was performed and, thus, no histology is available. However, this reference usually has a lower spatial resolution than approaches using visible epileptogenic lesions.

The aim of this study is threefold: (1) Foremost, to compare the MP2RAGE and MPRAGE sequences for the detection of cortical epileptogenic lesions at 3T using a state-of-the-art surface-based (SBM) processing pipeline–with a view to applying SBM also to ultra-high field MRI MP2RAGE data in the future. (2) Evaluating unsupervised surface-based machine learning classification procedures, because it may be hypothesized that epileptogenic lesions in visually MRI-negative cases have qualitatively different imaging characteristics than MRI-positive, and because the availability of MRI-positive subjects needed for training of supervised classifiers is often limited, e.g., when evaluating novel imaging contrasts or ultra-high field MRI. (3) Proposing a statistical evaluation for MRI-negative epilepsy cohorts with lobar hypotheses, based on simulating a random ‘guessing’ null-hypothesis process under an alternative free-response receiver operating characteristic (AFROC) paradigm.

## 2 Methods

### 2.1) Subjects, clinical data

We retrospectively included focal epilepsy patients who had undergone comprehensive workup for refractory epilepsy–including video-electroencephalography (EEG) telemetry and a standardized dedicated 3T MRI protocol that included MP2RAGE, MPRAGE and FLAIR sequences–at the epilepsy centres of the University Hospital Tübingen and the University Hospital Göttingen, Germany. Additionally, available MRI scans of healthy controls examined according to the same imaging protocol at the two centres were included.

In a clinical interdisciplinary (neurology, neuroradiology, neurosurgery, neuropsychology) case conference, a consensus hypothesis concerning the most likely seizure onset zone on a lobar level was formulated. For this, all available clinical information was used–video-EEG and MRI as well as detailed neuropsychological testing, FDG-PET (18 F fluorodeoxyglucose positron emission tomography) and intracranial EEG. Patients were designated MRI-positive if a lesion could be visually identified in MRI that was concordant with the clinical hypothesis. Otherwise, patients were termed MRI-negative and the lobe(s) of most likely seizure onset zone localisation was used for the evaluation (as explained further below).

Data were last accessed on 26 January 2022. During collection of clinical information at the two sites, some of the authors had access to information that could identify individual participants. After collection, all data were pseudonymized and MRI data from Göttingen was defaced (using the FSL deface software) [21] prior to transfer to Tübingen, where the analysis was carried out. Data collection was approved by the ethics committees at the University of Tübingen (project number: 738/2021A) and the University Medical Center Göttingen (project number: 16/10/17).

### 2.2) Imaging

Subjects were scanned on a 3T Siemens Magnetom Prisma MRI (Tübingen) or a 3T Siemens Magnetom Prisma fit (Göttingen), each with 64 channel head coils. The following 3D sequences with 1 mm isotropic resolution were acquired with very similar parameters at the two sites (see S2 Table in S1 File): (1) T1-weighted Magnetization-Prepared Rapid Gradient-Echo (MPRAGE). (2) T1-weighted Magnetization-Prepared 2 Rapid Gradient-Echo (MP2RAGE). (3) T2-weighted Sampling Perfection with Application optimized Contrasts using different flip angle Evolution-Fluid-Attenuated Inversion Recovery (T2-SPACE FLAIR). Visual quality control resulted in the exclusion of one control subject due to movement artefacts.

### 2.3) Image processing

#### 2.3.1) Preprocessing of MP2RAGE data (skullstripping)

‘Uniform’ MP2RAGE images [5] contain distinct ‘salt and pepper’ noise extracranially and in aerated sinuses, which is known to hamper processing with established segmentation and morphometry software optimized for T1-weighted contrast, such as Freesurfer. Workarounds include calculating ‘robust’ MP2RAGE images instead [22], in the process losing some of the homogeneity characteristics, and generation of a brain mask–using the raw proton density weighted image acquired at the second inversion time (INV2), a multiplicative image (uniform MP2RAGE and INV2) or the robust MP2RAGE [23, 24]–for skullstripping of uniform MP2RAGE data.

Here, as we were interested in the uncompromised intensity characteristics of MP2RAGE vs. MPRAGE for morphometry, we opted for a variant of the skullstripping method that we found to perform well in preliminary comparisons: The raw INV1 and INV2 images were input as separate channels into the Statistical Parametric Mapping (SPM 12, version 7771) unified segmentation framework [25] with default parameters. Then a skullstripping mask was generated by thresholding (level 0.5 for GM/WM, 0.99 for CSF), a logical ‘OR’ operation and morphological closing on the grey matter (GM), white matter (WM) and cerebrospinal fluid (CSF) tissue class probability maps. The resulting brain mask was applied to the ‘uniform’ T1-weighted MP2RAGE images.

#### 2.3.2) Surface-based feature extraction and augmentation

The widely adopted open-source software suite FreeSurfer (version 7.2.0) was used for cortical surface reconstruction and coregistration [26–28] based on T1-weighted MPRAGE and T1-weighted skullstripped uniform MP2RAGE images. For nine subjects’ MP2RAGE data, two initial control points per hemisphere had to be set manually because the automated processing failed, otherwise no edits were made and default settings were used.

The selection of surface features and augmentation steps was based on the typical radiological features of FCDs (cortical thickening, blurring of the gray-/white-matter boundary, subcortical FLAIR hyperintensity) as well as on previous literature concerning surface-based morphometry for the characterization and detection of epileptogenic lesions [12, 13, 19, 29, 30]. Overall, we aimed to first obtain a broad range of (augmented) surface features and then define different subsets of these features (see below) to evaluate how feature selection impacts performance.

Multiple morphometry features for each vertex on the reconstructed triangle mesh cortical surfaces were evaluated: Average convexity or sulcal depth (termed ‘sulc’) [27], mean curvature (‘curv’), cortical area and volume were output by the Freesurfer recon-all script by default. Cortical thickness [31] was estimated with a maximum of 15 mm instead of the preset 5 mm. The absolute Gaussian curvature (termed absolute intrinsic curvature index, ‘AICI’), similar to ‘local cortical deformation’ [29], was calculated after stripping outliers from raw Gaussian curvature data.

Furthermore, T1-weighted and FLAIR image intensities were sampled at various points along the surface normal (75%, 50%, 25% of cortical thickness, at the white-grey interface, 1 and 2 mm subcortically) for each vertex. To this end, FLAIR images were first coregistered to the respective T1-weighted volumes using boundary-based coregistration [32] and bias-field-corrected using the AntsN4 algorithm [33]. FLAIR data was not used for improving pial surface reconstruction in the Freesurfer pipeline, so as to enable assessment of the added value of FLAIR intensity information alone. Secondly, as raw signal intensities represent arbitrary values that depend on scanning parameters and processing, normalization is necessary to make the data amenable to inter-subject statistical analysis. Similar to normalized FLAIR signal intensity (nFSI) methodology [34], we re-scaled all T1-weighted and FLAIR values with the median of white matter intensities and the median of cortical intensities (determined by the Freesurfer segmentation) serving as per-subject references.

Subject surfaces were coregistered to a symmetrical template (fsaverage_sym) [35]. All measures were resampled to this template and smoothed with both 8 mm and 32 mm full-width at half-maximum (FWHM) Gaussian kernels. The difference between the 8 mm and 32 mm FHWM maps was calculated additionally, representing a Difference-of-Gaussians (DoG) filter–commonly used in image processing for edge and blob detection and feature extraction for machine learning (e.g. [36]), acting as a spatial band-pass that is straightforward to implement. Lastly, per-vertex asymmetry (i.e., the difference between hemispheres) was calculated for each original measure and smoothed with 8 mm FWHM. Related concepts (‘doughnut method’ in analogy to DoG filtering, asymmetry maps) have previously been applied to surface-based morphometry in epilepsy [29]. Overall, 76 (augmented) features were generated.

For the multivariate classifiers, different subsets of features were selected so as to study the impact of feature selection and augmentation methods: First, all 76 augmented/preprocessed features; second, a subset of all measures only at 8 mm FWHM smoothing without additional processed versions–as used for univariate GLM analysis; and third, a subset of six morphometry measures performing well in univariate analysis (cortical thickness, white/grey contrast, T1 and FLAIR normalized intensity at -1 mm subcortically and 50% of cortical thickness, each) at all smoothing and filtering levels (resulting in 24 features). In each respective case, an additional subset without any normalized FLAIR intensity measures was composed, so as to evaluate information conveyed by MPRAGE and MP2RAGE data exclusively. Overall, this resulted in six surface feature subsets.

Before input into the classifier pipelines, per-subject and subsequent per-vertex z-scoring of each measure was performed (as in [29]), in order to account for subject- and location-specific bias and variance, and as a simpler alternative to GLM modelling with covariates.

#### 2.3.3) Definition of lesion and hypothesis labels

For the lesional patients, one volume label was manually drawn for each lesion using all three available linearly aligned sequences and, in case of the 3 operated MRI-positive cases, also postoperative imaging for reference. These volume labels were projected onto both MPRAGE and MP2RAGE Freesurfer surface reconstructions, mapped to the symmetrical template and morphologically opened and closed to yield one continuous mask without holes. Due to slight variations in cortical surface reconstructions and coregistrations to the common template between MPRAGE and MP2RAGE data for each subject, the resulting surface labels in template space did not completely overlap (Jaccard-Index/intersection over union: mean 0.82, range 0.55–0.93). The intersection (logical ‘and’) of the two surface projections was representative for the combination of MPRAGE- and MP2RAGE-data and was used as a ‘strict’ label version for training of supervised classifiers (see below). Further morphological dilation by 4 vertices resulted in a ‘wide’ version used for automated detection performance evaluation (see below).

For the MRI-negative patients, lobar surface labels (based on the Desikan-Killiany cortical parcellation atlas [37]) were assigned based on clinical hypotheses and used for the detection performance evaluation.

### 2.4) Statistical analysis

#### 2.4.1) Direct comparison of MPRAGE/MP2RAGE segmentations and surface measures

We explored systematic differences between the MPRAGE and MP2RAGE Freesurfer segmentations and surface reconstructions. Here, we analyzed only control subjects. In a first step, three per-subject outputs of the volumetric segmentation (aseg)–total cortical gray matter volume, total cerebral white matter volume, brain segmentation volume–were compared using Wilcoxon signed-rank tests and visualized in boxplots and histograms of the paired differences. Secondly, we calculated vertex-wise paired (MPRAGE vs MP2RAGE) differences in cortical thickness. Surface overlays of the mean signed differences (across all control subjects) were rendered, and mean and standard deviation of absolute differences across hemispheres were calculated. This is the same methodology used in [24] so as to achieve comparability of results–except that we did not generate dedicated templates for each subject via the Freesurfer longitudinal stream but directly used the data coregistered and resampled onto the common surface space (fsaverage_sym). We also plotted and compared histograms of normalized T1-intensity sampled at 1 mm subcortically and at 50% of the cortical thickness.

#### 2.4.2) Univariate (GLM) statistical analysis

In a first step, vertex-wise univariate statistical analysis was performed with a subset of all surface-based morphometry (SBM) measures (only at 8 mm FWHM smoothing, 19 in total). A general linear model (GLM) was fitted for comparison of each subject with all controls (in case of controls, all remaining control subjects–as a ‘leave-one-out’ partitioning). Age, gender, scanner site and (for area, volume and thickness) estimated intracranial volume (eTIV) were included as nuisance covariates in the model. Z-scores (two-tailed) were used for detection performance evaluation (see below).

#### 2.4.3) Machine learning procedures

*2*.*4*.*3*.*1) Model selection*. Secondly, vertex-wise multivariate analysis was carried out using different machine learning approaches. As the dataset (which was compiled with availability of MP2RAGE data as the main criterium) contained only few MRI-positive lesional cases, this posed a significant limitation to training of supervised classifiers as performed in several other approaches[12, 13, 19]. Thus, we applied two unsupervised novelty detection methods that were trained only on control vertices:

First, we computed the Mahalanobis distance (MAH) of each vertex’s feature vector to the training set mean, based on robust mean and covariance estimates [38]. This approach would be expected to perform best if control vertex features were normally distributed, which is not the case in our sample (as assessed by Lilliefors-test p < 0.05, and visually q-q plots for individual features as well as q-q plot of squared Mahalanobis distance vs. chi squared distribution quantiles; not shown).

Hence, we also applied the isolation forest (IF) algorithm [39], an anomaly and novelty detection method with favorable classification performance with fixed parameters, and low computational requirements. For IF, we set the number of trees *t* = 100 and the subsampling size *ψ* = 2048 (a relatively high subsampling size was reported to be advantageous by the original authors for training on normal instances only).

For comparison, we also applied a supervised approach (in spite of the small training set). We chose a random forest classifier (RFC) [40], as established for biomedical pixel classification applications [36, 41]. Advantages of RFC include its performance, training speed, output of classification probabilities, and robustness with respect to hyperparameter choice. The latter aspect is especially advantageous because hyperparameter optimization usually relies on performance evaluation with random subsamples of data (i.e., k-fold partitioning or out-of-bag samples)–which is problematic for our application since significant data leakage can be assumed to occur between vertex-wise random subsamples due to the spatial smoothing of data during preprocessing. Subject-wise subsampling would be required, further reducing the small training set size. We set fixed hyperparameters at default empirically recommended values (number of trees = 100; number of variables to sample at each tree split = square root of total number of variables; maximum number of splits = number of observations– 1; minimum leaf size = 1; Gini’s diversity index as split criterion).

*2*.*4*.*3*.*2) Training and testing sets*. For the unsupervised approaches (MAH, IF), all control group vertices were used as the training set. For evaluation of the control subjects themselves (to assess specificity), only the vertices from all other control subjects were included in the training set, so that a separate model was fit for each control subject (in a leave-one-out scheme analogous to the univariate GLM analysis).

For the supervised RFC classifier, all control group vertices and all lesional vertices (from the MRI-positive cases) were used. Again, for the MRI-positive cases and the control subjects themselves, separate models were fit excluding the respective data in training (leave-one-out). In order to account for the class imbalance, a random subsample of all selected control group vertices (with a ratio of 1:10 of lesional to control vertices) were used for training.

For all classifiers, inference was performed on all vertices of each subject (left out from the respective training set) in turn. Mahalanobis distance is a positive real number, IF and RFC yield classification scores in the interval [0,^1^]. All maps were smoothed on the template surface with a Gaussian kernel of 4 mm FWHM so as to reduce small false-positive findings.

Evaluation of the outputs was not performed vertex-wise but in a clinically more meaningful way as described in the following section.

#### 2.4.4) Automated detection performance evaluation

*2*.*4*.*4*.*1) Alternative free-response ROC analysis*. GLM and classifier output surface maps were thresholded (see below for details) and clustered. A cluster was rated a ‘true positive’ if its centroid lay within the lesion or hypothesis label (which is a more stringent criterion than applied in many other studies, see discussion), or otherwise, a ‘false positive’. From these ratings, the ‘true positive fraction’ (TPF, number of detected labels divided by the total number of labels, similar to sensitivity) the ‘false positive fraction’ (FPF, number of control subjects with any false positive cluster divided by total number of control subjects, similar to specificity) and the ‘false positive rate’ (FPR, mean number of false positive clusters in control subjects) were determined.

Computing these performance figures over a range of thresholds or operating points could yield free-response receiver operating characteristic curves (FROC, when plotting TPF against FPR) and alternative FROC (AFROC) curves (when plotting TPF against FPF [42, 43]). We used the latter in this project because the area under the AFROC curve (AUC) is bounded and can serve as a summary statistic for prediction performance. It would be extremely computationally expensive to evaluate all possible thresholds; we aimed to sample thresholds as densely as practically feasible as to minimize a possible impact on the estimation on AFROC AUC. It should be noted that only false positive findings in control subjects were taken into account, not those in subjects with lesions or hypotheses, because multiple lesions may be present in patients [44]. Similar (alternative) free-response ROC methodology was applied for voxel-based morphometry in epilepsy before [9, 13, 45].

*2*.*4*.*4*.*2) Assessing statistical significance of AFROC AUCs*. In classical ROC analysis for binary classification, a random ‘guessing’ process can serve as a theoretical lower bound and null hypothesis for comparing diagnostic performance: In this case, sensitivity equals specificity for any threshold, so that the guessing ROC curve is a diagonal line with an AUC of 0.5. In a free-response task, a null guessing process places lesion marks in random locations–in our case, anywhere on the cortical surface with a random uniform distribution. Thus, the null hypothesis for a lesion detection process is that its probability of placing a lesion mark is equal for every vertex on the cortical surface.

**Fig 1:**
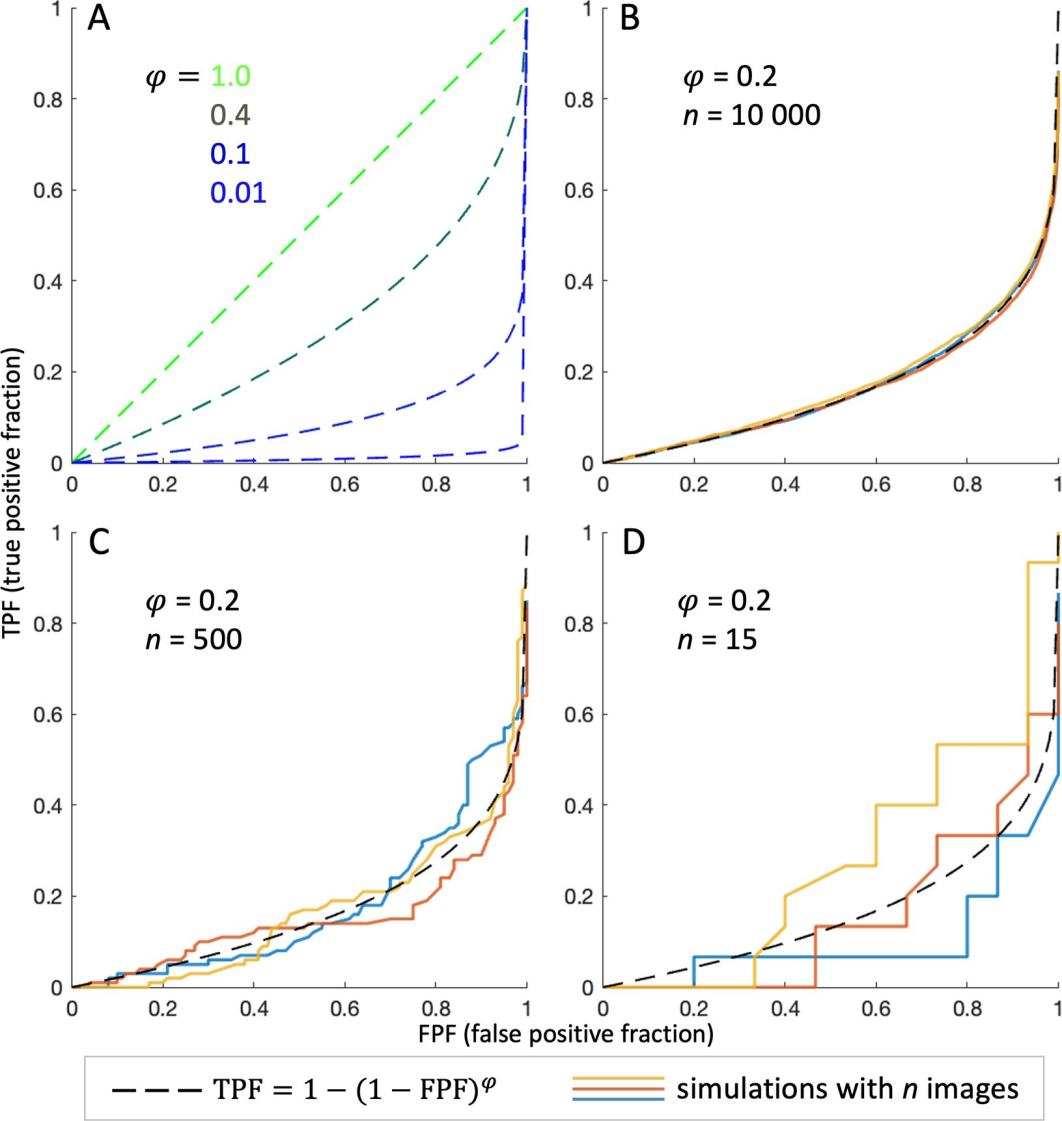
(A) Modeled alternative free-response receiver operating characteristic (AFROC) curves of a random ‘guessing’ process for different relative ground truth lesion label sizes *φ*. It can be appreciated that for *φ* = 1, TPF = FPF and AUC = 0.5 (the ground truth labels cover the entirety of every ‘lesional’ subject, the task effectively becomes a binary classification as in classical ROC analysis). Contrarily, for *φ* → 0, guessing AUC → 0. (B-D) Simulated random process AFROC curves for *φ* = 0.2 and different numbers of subjects *n*, overlaid onto the model (3 curves each). It is visible that for lower *n*, the distribution broadens.

AFROC AUC as a summary statistic for lesion detection performance (as described in the previous paragraph) under this null hypothesis depends on the size of the ground truth lesion labels: the larger the ground truth, the more likely there will be concordant random marks. In our MRI-negative cohort, the ground truth labels cover ≈ 20% of the cortical surface (see results), so that this effect in AFROC evaluation is not negligible. For very large numbers of examined subjects, AFROC curves under the null hypothesis of random lesion mark placement can be modeled (see S1 Methods in S1 File) as TPF = 1 - (1—FPF)*^φ^*, and $\text{AUC}=\frac{\varphi }{\varphi +1}$, where *φ* is the ratio of ground truth lesion label area to total subject surface area. For illustration, we plotted AFROC curves assuming different *φ* ranging from 0.01 to 1 (Fig 1A). For cohorts with a finite number of subjects *n*, a null hypothesis guessing process produces a distribution of AFROC curves (and resulting AUCs) that approaches the function stated above for large *n* and broadens with decreasing cohort size, as we verified using a simplified simulation (reduced number of vertices, *φ* = 0.2 for all subjects; Matlab code available), see Fig 1B–1D.

We are not aware of an analytical description of the null distribution of AFROC AUCs for a given cohort size and for differently sized ground truth labels. Thus, to obtain AFROC AUC distributions under the null hypothesis for MRI-positive and MRI-negative cohorts in this work, we resorted to a Monte Carlo simulation: In each run of the simulation, lesion marks were placed randomly under the null hypothesis (equal probability for a lesion mark at each vertex) and the resulting AFROC AUC for the cohort was evaluated. A large number of simulation runs yielded a distribution of AFROC AUC under the null hypothesis. For a given detection approach *D*, the resulting AFROC AUC_*D*_ could then be compared to the Monte Carlo null hypothesis distribution as a test statistic in a one-sided test, resulting in a p-value [46]: When *m* is the number of total simulated Monte Carlo AFROC AUC statistics, and *b* the number of statistics greater or equal to AFROC AUC_*D*_, then *p*_*D*_ = (*b* + 1)/(*m* + 1). We performed 10^5^ simulation runs, resulting in a minimum estimated p-value of approximately 10^−5^.

We performed these tests for all univariate (GLM) and multivariate lesion detection approaches described above. We applied a Bonferroni correction as a conservative method to limit the family-wise error rate at the 5% level.

Statistically significant effects in these group analyses may be conveyed by only very few subjects in a larger cohort. As a sensitivity analysis, we also assessed detection significance per-subject: The strictest threshold that allowed detection of a given subject’s lesion was determined, the FPF at this threshold level was termed FPF_detection_ for that subject. Then, the probability that a ground truth label of the same relative size *φ* would be detected under the random null hypothesis is (again) given by *P*(FPF_detection_) = 1-(1-FPF_detection_)*^φ^*.

### 2.5) Software

All data analysis, image processing and visualization was performed using Freesurfer (version 7.2.0, https://surfer.nmr.mgh.harvard.edu/) and Matlab R2021b (Mathworks, Natick, Massachusetts, USA), some steps were parallelized on a local Sun Grid Engine (version 6.2u5). The locally developed code is available at github.com/ckronlage/epi_SBM_scripts_pub under a GPL license.

## 3 Results

### 3.1) Subjects and clinical data

We complied a dataset comprising 32 focal epilepsy patients (21 from Tübingen, 11 from Göttingen; 56% female, mean age 30.9 years ± 11.5 SD)) and 94 healthy controls (Tübingen 38, Göttingen 57; 51% female, mean age 28.8 ± 8.0 years) with standardized MRI images available for all subjects (MP2RAGE, MPRAGE and FLAIR).

For the epilepsy patients, the following additional investigations were performed and considered for formulating a hypothesis concerning seizure onset zone: Neuropsychological testing (in 30/32 patients), FDG-PET (19/32) and intracranial EEG (9/32 patients). Surgery was performed in 5/32 patients, with findings upon histopathology in 2/5 patients (FCD IIb in both) and clinical follow-up information was in 4/5 (Engel class I for all 4).

Of the 32 patients, 5 were classified as ‘lesional’ or MRI-positive (4 patients with suspected FCD, 2/4 histopathologically confirmed) and one a suspected subtle dysembryoplastic neuroepithelial tumour (DNET). The remaining 27 patients were MRI-negative, also upon repeat review. Clinical details for all patients are stated in S1 Table in S1 File.

### 3.2) Direct comparison of MPRAGE/MP2RAGE segmentations and surface measures

**Fig 2:**
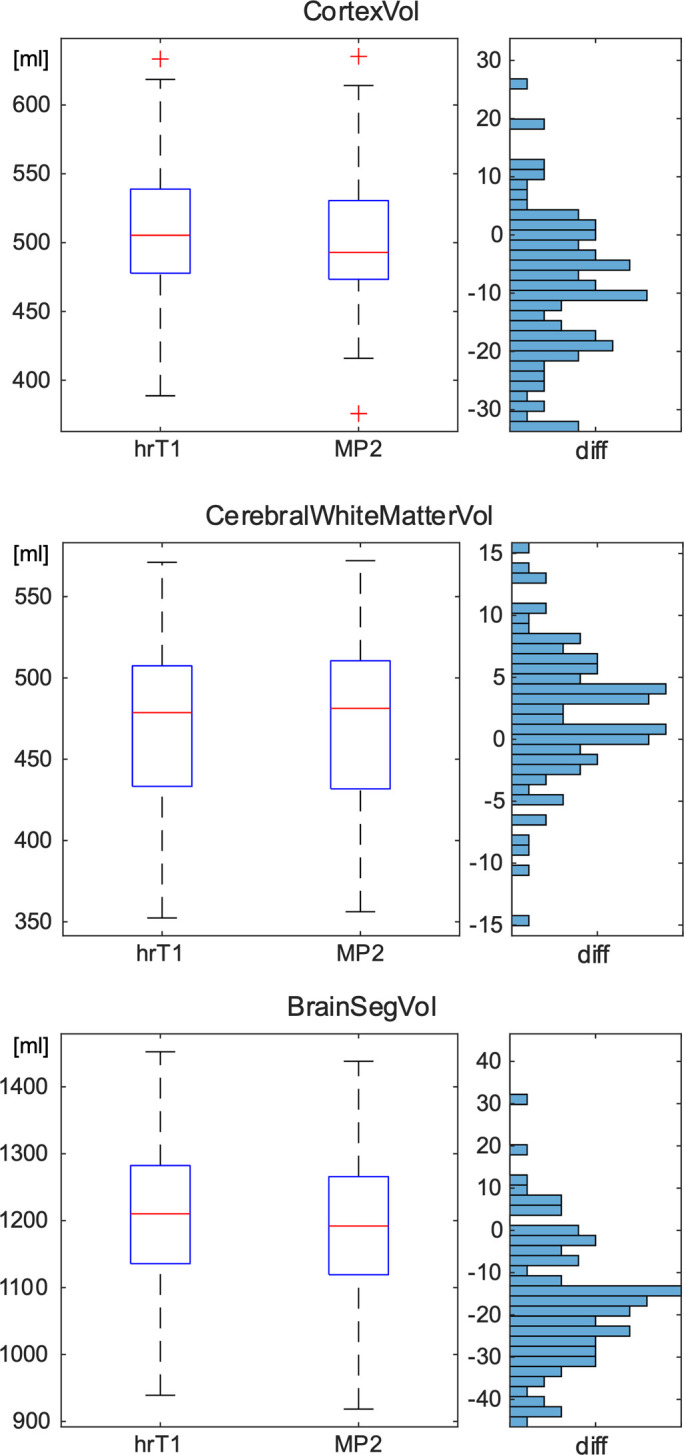
Comparison of per-subject volumetric measures (total cortical gray matter volume, total cerebral white matter volume, brain segmentation volume) between MPRAGE and MP2RAGE scans. It becomes apparent that MP2RAGE results in slightly smaller cortex and brain segmentations. Boxplots show absolute distributions, histograms paired differences (MP2RAGE-MPRAGE). All units are milliliters. In all three cases, group differences are statistically significant (Wilcoxon signed rank paired test p < 0.01).

In pairwise comparisons of Freesurfer segmentations (Fig 2), cortical volume and overall brain volume estimates were slightly smaller in MP2RAGE than in MPRAGE, white matter was slightly larger.

The average absolute thickness difference across all 94 control subjects’ 188 hemispheres was 0.330 mm (standard deviation 0.032 mm).

**Fig 3:**
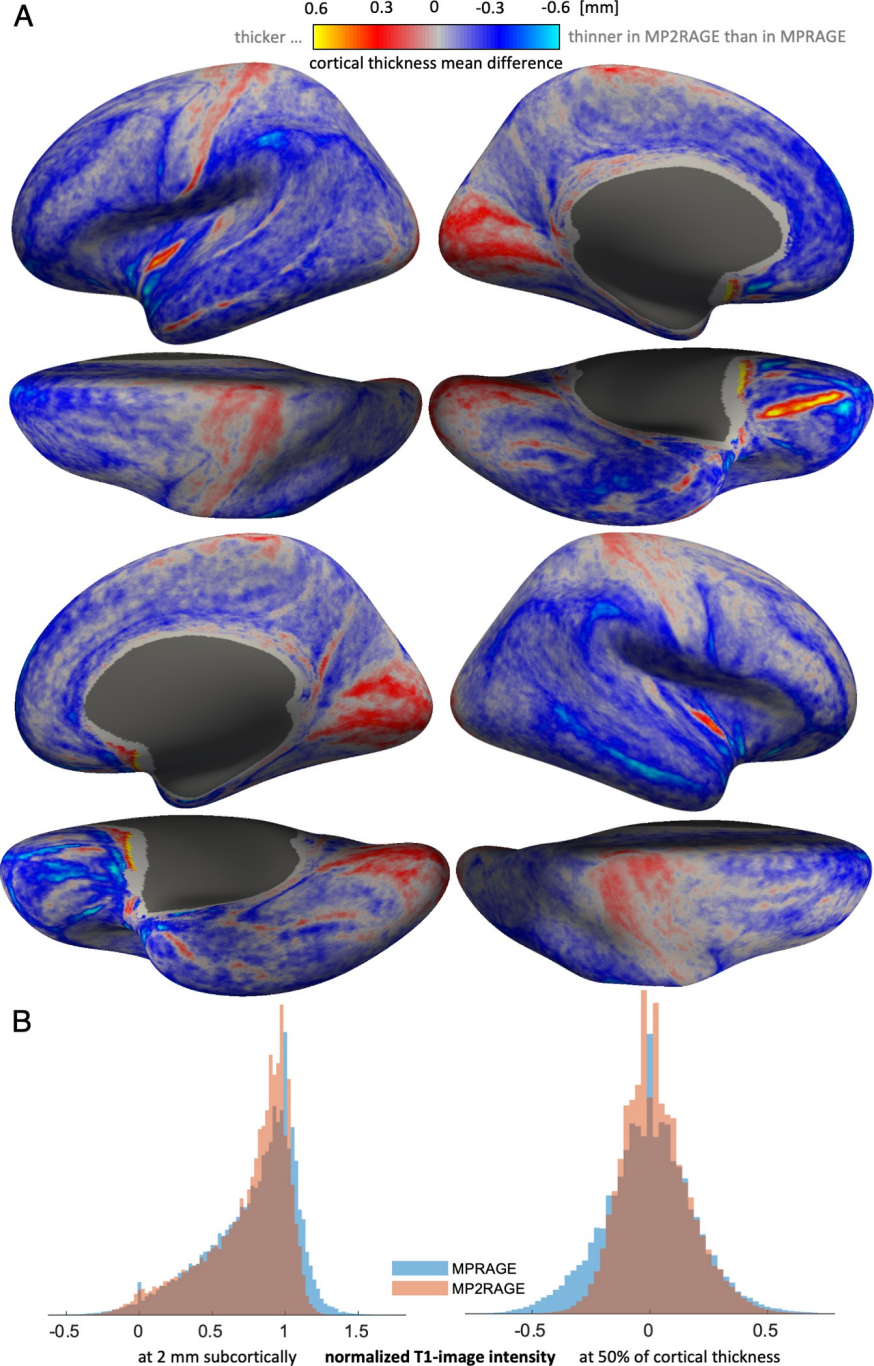
(A) Mean vertex-wise paired differences of cortical thickness between MPRAGE and MP2RAGE reconstructions–in control subjects–rendered on the template inflated cortical surface. Overall, the cortex was estimated thinner based on MP2RAGE. Large deviations appear in the basal frontal lobe, which may be due to misclassification of dura due to extracerebral noise in MP2RAGE. The cortex is reconstructed thicker in the central and medio-occipital regions, corresponding to the ‘unimodal’ primary sensory, motor and visual areas–a pattern that resembles and may be explained by different cortical myelination (see discussion). (B) Histograms of normalized T1-image intensity in all control vertices, sampled at 2 mm subcortically (left) and mid-cortically (right), for MPRAGE and MP2RAGE. MP2RAGE intensity distributions are narrower, likely a result of advantageous bias-field correction characteristics of MP2RAGE.

Maps of mean differences in cortical thickness between MPRAGE and MP2RAGE (Fig 3A) show that cortex is estimated thinner in MP2RAGE, overall. Some regions such central and medio-occipital, however, are estimated thicker on average in MP2RAGE.

We also compared histograms of normalized T1-intensity sampled in MPRAGE and MP2RAGE volumes at 1 mm subcortically and mid-cortically (Fig 3B). Besides microstructural gradients (i.e., differences in myelination) [47], variations of cortical intensity are caused by technical factors such as image inhomogeneity, partial volume effects and segmentation quality. Normalized T1 image intensities obtained from MP2RAGE show a narrower distribution.

### 3.3) Null hypothesis simulations and AFROC evaluation

**Fig 4:**
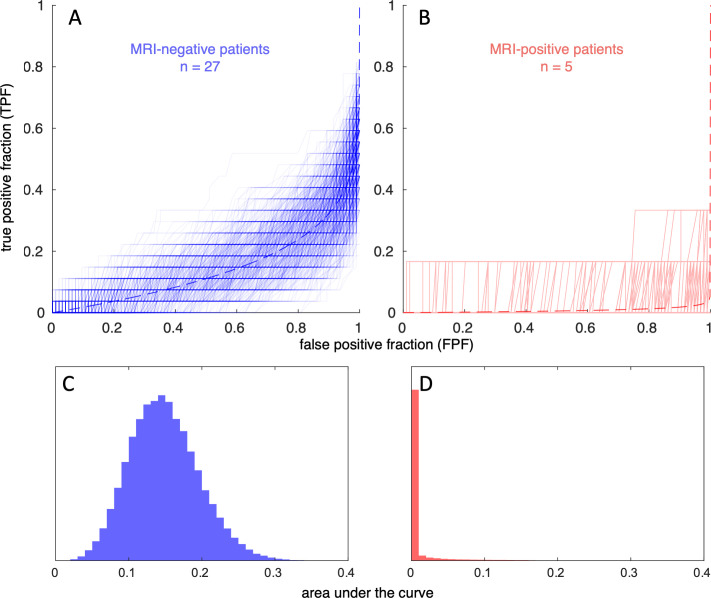
(A, B) Overlay of simulated ‘guessing’ AFROC (alternative free-response receiver operating characteristic) curves based on the ground truth lesion label characteristics in our cohorts. Dashed lines show a least-mean-squares fit of the exponential model function TPF = 1-(1-FPF)*^φ^* to all data. (C, D) Histograms of AUCS (areas under the AFROC curves) for 10^5^ simulations. (A, C) refer to our MRI-negative and (B, C) to the MRI-positive subjects.

The distribution of AFROC AUC under the null hypothesis (i.e., the apparent lesion detection performance of a random guessing process) depends on the size of the ground truth lesion labels. The lobar hypothesis labels defined for our MRI-negative patients covered a significant fraction of the total cortical surface (*φ*, mean 0.19, range 0.02 to 0.36) in comparison to the MRI-positive patients (mean 0.007, range 0.002 to 0.016). In consequence, the null hypothesis AFROC AUC distribution in the MRI-negative cohort differed substantially (mean 0.14) from the lesional cohort (mean 0.01), see Fig 4C and 4D. The depicted null distributions were used for evaluating the different surface-based morphometry lesion detection approaches.

### 3.4) Univariate GLM analysis lesion detection performance

**Fig 5:**
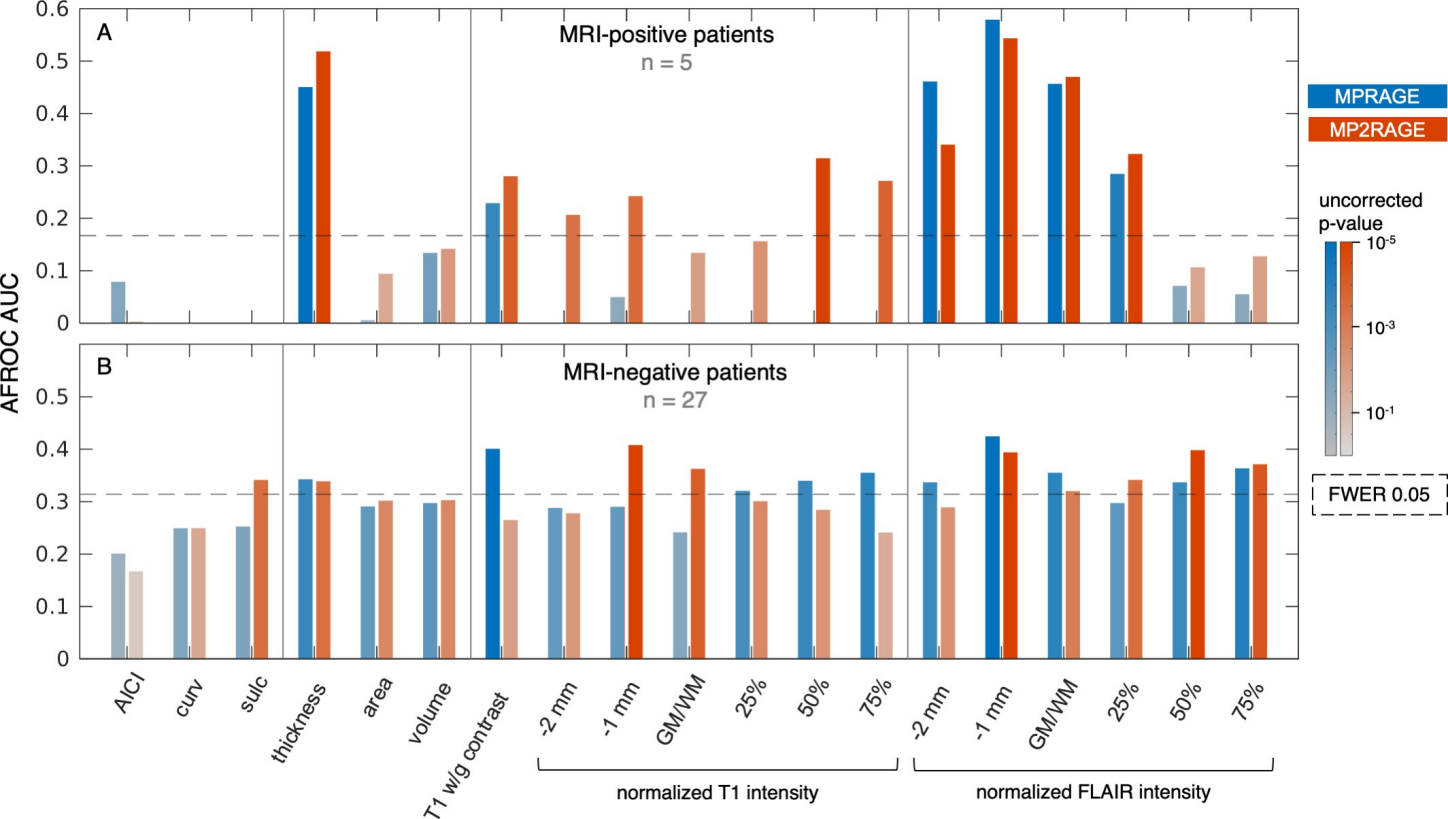
Results of the automated detection performance evaluation for univariate surface measure analysis, summarized by the area under the alternative free-response ROC curve (AFROC AUC). (A) MRI-positive patients, (B) MRI-negative cohort with electroclinical lobar hypotheses, compared between MPRAGE- (blue) and MP2RAGE-based (red) data. The color scale depicts the uncorrected p-value for each AUC under the simulated null distribution. The dashed line reflects the p-value/AUC threshold for a family-wise error rate (FWER) of 0.05 with Bonferroni correction for 38 comparisons in each cohort.

Results varied substantially according to the respective surface measure employed for univariate vertex-wise analysis. For the MRI-positive patients (Fig 5A), the best lesion detection performance both with MPRAGE and MP2RAGE data was obtained using cortical thickness (AUC 0.45 [MPRAGE] and 0.52 [MP2RAGE], difference +0.07), white/grey contrast (0.23 / 0.28 / +0.05) and subcortical FLAIR intensity measures (at 1 mm subcortically 0.58 / 0.54 / -0.04). In contrast, analysis of normalized T1 weighted image intensity yielded a statistically significant detection performance only with MP2RAGE data.

In the MRI-negative cohort (Fig 5B), the same trend but also some different associations were observed: In addition to cortical thickness and FLAIR intensity measures, notably, MP2RAGE sulcal depth and MPRAGE cortical normalized intensities enabled lesion detection concordant with the lobar hypotheses to a statistically significant degree. Some measures that exhibited good performance in the MRI-positive cohort such as MP2RAGE white/grey contrast and MP2RAGE cortical normalized intensity did not reach significance in the MRI-negative group. Overall, the best AUCs in the MRI negative cohort were much lower (0.40 for MPRAGE white/grey contrast, 0.41 for MP2RAGE normalized T1 intensity at 1mm subcortically).

### 3.5) Multivariate classifier lesion detection performance

We used different sets of surface measures as input for the training and classification methods and found that the computed detection performance varied considerably.

**Fig 6:**
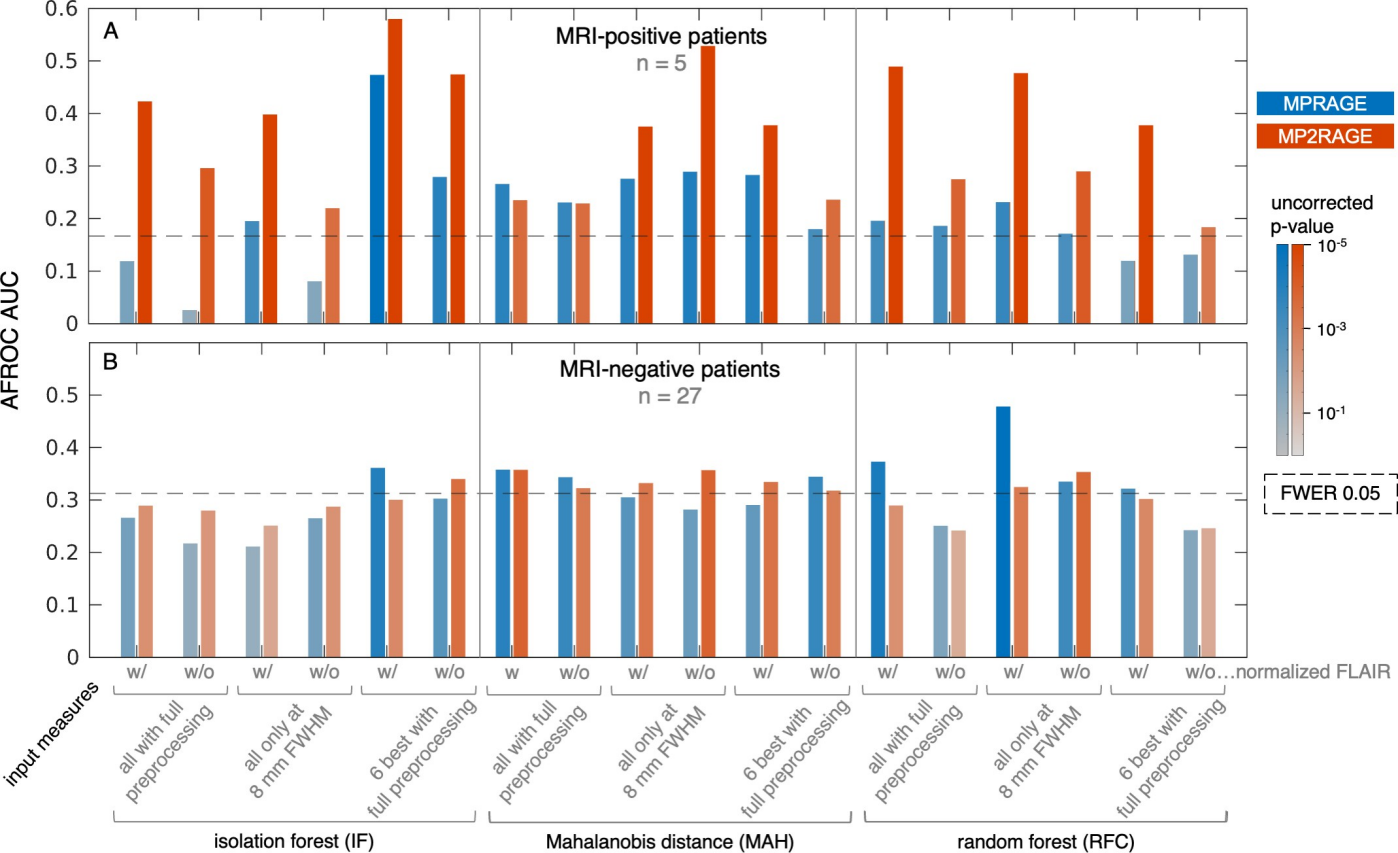
Results of the automated detection performance evaluation for unsupervised (IF: Isolation forest, MAH: Mahalanobis distance) and supervised (RFC: Random forest) classifiers, based on different sets of surface measures each with (w/) or without (w/o) FLAIR intensity data. Performance is summarized by the area under the alternative free-response ROC curve (AFROC AUC, y-axis). (A) MRI-positive patients, (B) MRI-negative cohort with electroclinical lobar hypotheses, compared between MPRAGE- (blue) and MP2RAGE-based (red) data. The color scale depicts the uncorrected p-value for each AUC under the simulated null distribution. The dashed line reflects the p-value/AUC threshold for a family-wise error rate (FWER) of 0.05 with Bonferroni correction for 36 comparisons in each cohort.

In the MRI-positive patients (Fig 6A), the best performance of the supervised random-forest classifier (RFC) was achieved using the larger sets of measures, with maximum AUCs of 0.23 (MPRAGE) and 0.49 (MP2RAGE). The additional preprocessing of surface measures (asymmetry, two smoothing levels, difference of Gaussians filtering) did not result in a major increase in detection performance in this dataset. In comparison to the best results obtained in univariate GLM-based analysis, the RFC pipeline yielded lower AUCs for MRI-positive patients.

**Fig 7:**
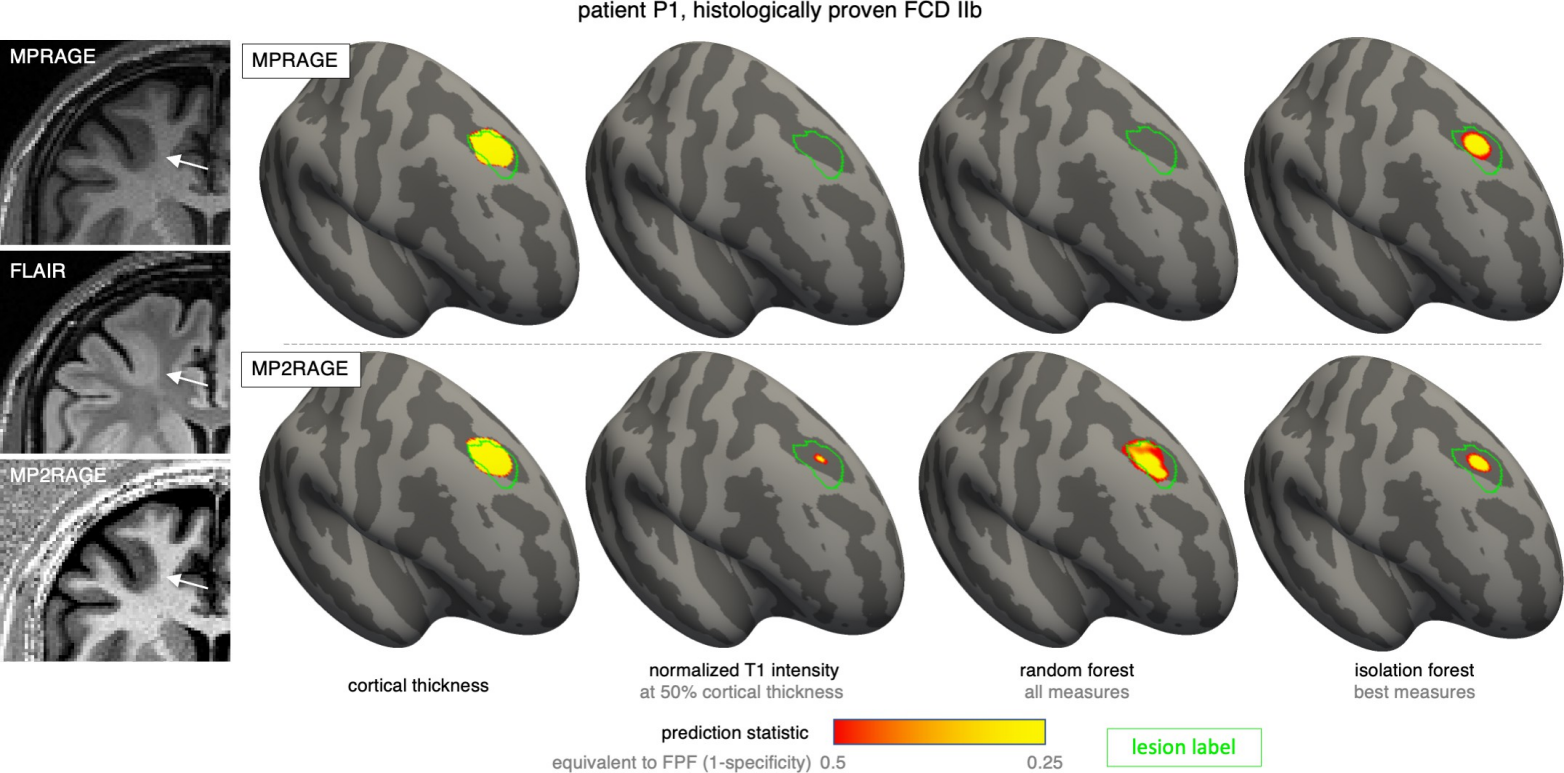
Exemplary lesion detection results in a patient with histologically proven FCD IIb. Left: Coronal views of the original image data showing the lesion. Right: Overlays of the prediction statistic on the inflated template surface for different selected univariate and multivariate approaches. The colorscale is aligned according to the false-positive fraction in each case. The green outline depicts the border of the manually defined ground truth label. It can be appreciated that the MP2RAGE outperforms MPRAGE, and that whereas different approaches all allow detection of the lesion, simple univariate analysis of cortical thickness performs best in this case.

For the unsupervised classification approach detection based on evaluation of Mahalanobis distance (MAH), we observed higher and statistically significant detection AUCs with all input measure sets. For isolation forest (IF), performance was best using the smallest set of most relevant surface measures (AUC 0.47 [MPRAGE], 0.58 [MP2RAGE], +0.11 [diff] with FLAIR; and 0.28 [MPRAGE], 0.47 [MP2RAGE], +0.19 [diff] without FLAIR). In nearly all cases for all classifiers, detection performance based on MP2RAGE data exceeded MPRAGE. Fig 7 shows exemplary detection results in an MRI-positive patient.

In the MRI-negative cohort (Fig 6B), best detection AFROC AUCs and corresponding p-values were lower than for MRI-positive patients, with some–such as RFC using measures only at 8 mm FWHM smoothing–exceeding the significance threshold with conservative Bonferroni-correction. The supervised RFC classification when using a reduced set of features from MPRAGE data exhibited a comparatively high performance (AUC 0.48). There was no general trend of higher performance in MP2RAGE than in MPRAGE.

### 3.6) Assessment of results on the single-subject/lesion level

In order to assess whether the described significant detection results in the MRI-negative cohort might be attributable to large effects in only a single or a few subjects, we computed detection p-values for all individual lesions (based on the strictest threshold at detection as well as the size of the hypothesis label as described in the methods), results are shown as heatmaps in S1 Fig in S1 File. A pattern with only few extreme cases likely conveying a group effect was apparent for some detection approaches (e.g., MPRAGE isolation forest approaches), in others we could not identify an according pattern (e.g., MPRAGE T1 white-grey contrast, MP2RAGE -1 mm normalized T1 intensity, etc.).

**Fig 8:**
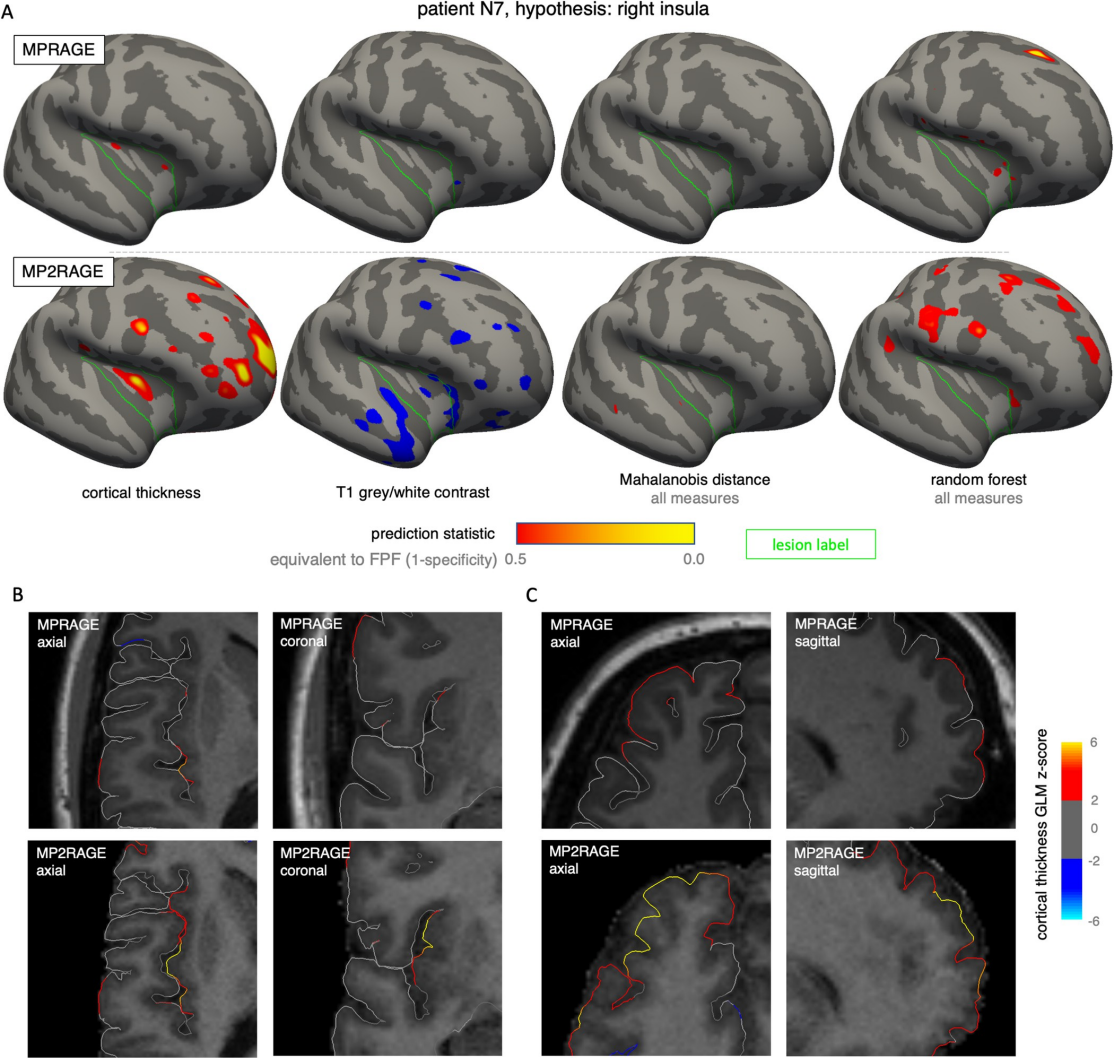
Exemplary lesion detection results in an MRI-negative patient. (A) Overlays of the prediction statistic on the inflated template surface for different selected univariate and multivariate approaches. The colorscale is aligned according to the false-positive fraction in each case. The green outline depicts the border of the lobar hypothesis label. (B, C) Original image data with overlaid tracings of the reconstructed pial surface, colored according to the cortical thickness GLM z-score, showing the maximum value in the temporal hypothesis label (B) and frontopolar (C). Overall, the results agree well with the clinical constellation in this patient: Whereas the hypothesis was formally defined as right insular, upon intracranial EEG also seizures originating from right frontal were recorded; in the end, the multifocal localization contraindicated surgery.

Upon closer examination of the most extreme findings in the single-lesion analysis, some appeared to represent actual potential lesion findings (Fig 8), whereas others were likely caused by processing artefacts (S2 and S3 Figs in S1 File).

## 4 Discussion

In this study, we compare MP2RAGE and MPRAGE for the detection of epileptogenic lesions using a surface-based morphometry pipeline and find that MP2RAGE generally performs at least on par with the conventional T1-weighted MPRAGE sequence.

A previous comparison of Freesurfer cortical surface reconstructions between the two sequences [24] found systematically lower cortical thickness estimates for MP2RAGE. We calculated a mean per-vertex thickness difference of 0.330 mm, which is comparable to results reported by Fujimoto and coworkers (MP2 vs. MEM repeat 0.307 ± 0.017 mm; rescan 0.298 ± 0.013 mm). A systematic difference in cortex segmentation based on MP2RAGE data may be explained by how partial volume effects are modeled [48].

Furthermore, we observe a distinct pattern of a thicker cortex in the central and medio-occipital regions, corresponding to the ‘unimodal’ primary sensory, motor and visual areas. We are not aware that this has been described before for MP2RAGE cortex segmentations. The pattern strikingly resembles–and thus might be explained by–variations in cortical myelination as apparent on MRI by means of T1w/T2w ratio imaging and quantitative T1 mapping [47, 49].

Besides systematic differences in segmentation results, segmentation reproducibility has also been described to be slightly lower for MP2RAGE [24]. In our dataset, segmentation errors at the pial surface occurred more frequently in MP2RAGE data (as assessed visually), despite skullstripping prior to processing in Freesurfer, which may contribute to differences in cortical surface reconstruction.

Whereas segmentation errors may affect lesion detection in some cases (S2 Fig in S1 File), overall, we observed a slightly better performance of MP2RAGE compared to MPRAGE in the MRI-positive patients based on univariate analysis of cortical thickness. Similar results favoring MP2RAGE were also reported for lesion detection in epilepsy using VBM [8, 9].

FLAIR hyperintensity is a characteristic feature of FCDs, in particular for type IIb, and widely utilized for automated lesion detection in epilepsy [20, 29, 30, 50]. Unsurprisingly, we find that univariate evaluation of subcortical FLAIR image intensities exhibits good performance for the detection of MRI-positive epileptogenic lesions.

On the other hand, for the detection of FCDs, the intensity of T1-weighted images has only rarely been evaluated explicitly–though sometimes indirectly in the form of T1-weighted image white-grey contrast. Yet, subcortical T1 intensity has been shown to be reduced in FCD [30]. Here, we systematically assess the utility of normalized T1-weighted image intensity sampled in the cortex and subcortically. In the MRI-positive patients, MPRAGE conveys no information using these measures, whereas MP2RAGE exhibits significant detection performance when sampling intensities both subcortically and in the middle and superficial cortex.

The application of supervised machine learning classifiers can be considered state-of-the-art for multivariate surface-based morphometry in epilepsy [12, 13, 19, 29]. However, supervised classifiers require large training samples that include lesional cases. In some circumstances, the amount of available data is fundamentally limited, such as in the evaluation of novel imaging contrasts or in ultra-high field MRI. Correspondingly, this study included only a small set of six delineated lesions in five subjects. Additionally, lesions in visually MRI-negative cases may exhibit different characteristics in terms of histopathology (as discussed below) and consequently also imaging. Thus, it is debatable whether supervised classifiers trained on MRI-positive cases (often FCD type II) are well suited to identify epileptogenic structural abnormalities in MRI-negative cases.

Various unsupervised (‘one-class learning’, ‘novelty-detection’) machine learning approaches have been developed, which require only normal samples for training. We applied two such unsupervised learning approaches: Estimation of Mahalanobis distance (MAH), which implicitly relies on the normality of the multivariate feature distribution, and isolation forest (IFST) [39], an algorithm described to perform well on complex and high-dimensional data. Because unsupervised novelty detection is known to suffer from ‘the curse of dimensionality’, i.e., a reduction in performance caused by irrelevant features, we evaluated these classifiers on different selected subsets of input measures.

Indeed, among the different unsupervised classifiers, the IFST algorithm using a reduced subset of input features performed best. In comparison, this yielded a AFROC AUC in MRI-positive subjects comparable to univariate analyses of the most meaningful features. For comparison (even though the set of MRI-positive lesions available for training was very small, six lesions in five subjects), we also applied a supervised classifier (RFC). Expectedly, this approach exhibited its best performance with the maximum number of input features, but overall did not perform as well as the unsupervised classifiers or univariate analyses in the MRI-positive subjects (see Figs 5 and 6).

These results suggest that unsupervised novelty detection may represent a viable approach for detecting epileptogenic lesions in situations where the amount of data available for training is limited because acquisition is very costly, like in ultra-high field MRI.

Our group of MRI-negative patients with only lobar hypotheses is particularly challenging, because it is not possible to define precise ground-truth labels. Still, this is the most clinically relevant group in which to apply such a diagnostic tool. Here, we propose a statistical method of assessing whether a given detection method significantly outperforms a null-hypothesis guessing process. Another group recently used a region-wise binary classification to assess whether findings of voxel-based morphometry in MRI-negative focal epilepsy were statistically significant [51]. Our approach is similar but also takes into account the different sizes of these regions.

It can be hypothesized that epileptogenic lesions in MRI-negative focal epilepsy patients might exhibit qualitatively different morphological abnormalities on MRI, and do not simply represent quantitatively more subtle cases of MRI-positive lesions such as FCD type II. In truly (and not ‘ever reported’) MRI-negative cases, the characteristics of underlying lesions can only be derived from histopathology. In the literature, postoperative diagnoses in MRI-negative focal epilepsy include FCD type I, ganglioglioma, gliosis, hamartia, cavernoma and mild malformations of cortical development (mMCD) with oligodendroglial hyperplasia in epilepsy (MOGHE), [2, 51–53]. In a part of these cases, there might not even be any detectable structural abnormality underlying epileptogenicity: Even epilepsy surgery without histopathological findings can lead to a favorable outcome and seizure freedom. The two MRI-negative patients in our cohort who had undergone epilepsy surgery also achieved Engel class I outcome even though histopathology was without pathological findings.

The results obtained from our MRI-negative cohort are striking insofar as univariate analyses of cortical thickness and subcortical FLAIR intensities, the most relevant features in the MRI-positive cohort, perform relatively poorly in the MRI-negative cohort in comparison. Similarly, in a cohort from a different epilepsy center, the statistical concordance of voxel-based morphometry (MAP18) findings (mainly based on the features cortical thickness and gray/white-matter junction sharpness) in truly MRI-negative patients was found to be limited [51]. In contrast, in this work, some surface measures such as sulcal depth, cortical MPRAGE (but not MP2RAGE) T1-weighted image intensities and superficial cortical FLAIR intensities appear to yield significant localizing information, whereas this does not apply at all to MRI-positive lesions. This would suggest that different morphological and intensity features might be sensitive to MRI-negative epileptogenic lesions, however it does not align with clinical experience (i.e., if sulcal depth alone were highly predictive of epileptogenic lesions, it would be easily visible). Furthermore, contrary to what we had hypothesized, the unsupervised machine learning methods employed in this work did not exhibit any convincing performance in the MRI-negative cohort. Overall, our data do not allow new conclusions concerning a hypothetically distinct imaging profile of MRI-negative epileptogenic lesions.

In absolute numbers, our detection results (best AFROC AUC of 0.58 in MRI-positive patients, 0.48 in the MRI-negative cohort) are much worse than what has been reported by other groups (e.g., AUC of 0.75 [12, 13, 15, 19, 54]) using similar surface-based methods.

We attribute this, firstly, to the different cohorts studied: In this study, only few MRI-positive patients could be included, whereas the MRI-negative patients were not defined as ‘ever MRI negative’ with visible lesions upon repeat review, but represented ‘hard’ cases, largely without surgery and thus postoperative histology. Using a similar dataset (collected from one center only), our group previously performed voxel-based morphometry (VBM) and achieved a maximum AUC of only 0.39 [9]. A group from Munich recently applied a slightly different statistical approach that also showed a lesion ‘detection’ performance of VBM that hardly exceeded random guessing in truly MRI-negative patients [51].

Secondly, the criteria for lesion detection in this study were defined more strictly: Only if a cluster centroid lay within the lesion label, it was counted as a true positive. In comparison, many studies require only a certain amount (e.g., one third of the cluster [9, 45]) of overlap between the cluster and the lesion label or even count any overlap at all as a ‘detection’ [12, 13, 15, 19]. We chose the stricter criterion because the reduction of clusters to centroid points facilitated the simulation of a random guessing null-hypothesis process. We would also argue that this definition is useful in that it brings the methodology in line with ‘(alternative) free-response ROC’ as a well-described statistical paradigm [42, 43]; whereas ROC analyses have been conducted by various groups in epilepsy lesion detection, the connection to this term has not been explicitly made, as far as we are aware.

Strengths of this work include the high-quality MRI dataset studied (three sequences throughout, acquired on almost identical scanners at two sites), the state-of-the-art surface-based postprocessing applied, and the critical statistical assessment of results. The conclusions that can be drawn from this study are mainly limited by the small size of the dataset and, more specifically, the small number of patients in the MRI-negative group having undergone intracranial EEG and epilepsy surgery, which would represent the diagnostic gold standard in MRI-negative focal epilepsy. However, our MRI-negative cohort is representative of clinical practice, where only a minority of MRI-negative focal epilepsy patients proceed to surgery (e.g., 15% [2]). A number of previous works have studied similar cohorts with largely clinical ground truth hypotheses [9, 45, 55]. As truly MRI-negative patients are the clinically most relevant cases for improving lesion detection by imaging, in this work, we aim to make coarse clinical ground truths more usable by proposing a novel statistical approach for detection performance evaluation.

In conclusion, this study shows that, at 3T, MP2RAGE for surface-based morphometry in epilepsy is at least comparable to MPRAGE and that analysis of MP2RAGE uniform image intensities may provide additional diagnostic information. In the future we will investigate the utility of surface-based morphometry using MP2RAGE acquired at ultra-high field. Given the sample size limitations of such studies, we furthermore demonstrate that unsupervised novelty-detection machine learning approaches may be useful for the detection of epileptogenic lesions when there is only a limited lesional training set available. Third, we propose a statistical method of assessing lesion localization performance in MRI-negative patients with lobar EZ hypotheses.

## Supporting information

S1 File (PDF)
