# Supplementary material for: MP2RAGE vs. MPRAGE surface-based morphometry in focal epilepsy
Source: PLoS One. 2024 Feb 8;19(2):e0296843. doi: 10.1371/journal.pone.0296843 (PMC10852321; doi:10.1371/journal.pone.0296843)
Supplement: S1 File — (PDF) [file pone.0296843.s001.pdf]

MP2RAGE vs. MPRAGE surface-based morphometry in focal epilepsy

Cornelius Kronlage[1], Ev-Christin Heide[2], Gisela E Hagberg[3,4], Benjamin Bender[5], Klaus Scheffler[3,4], Pascal Martin[*1], Niels Focke[*1,2]

Supplementary material

S1 table: Patient characteristics. ID: numbered MRI-positive (P…) and MRI-negative (N…) cohorts. Localizing information – L (left), R (right), F (frontal), C (central), P (parietal), T (temporal), I (insular), O (occipital). FCD (focal cortical dysplasia). DNET (dysembryoplastic neuroepithelial tumour).

ID	age	sex	age of onset	MRI (suspected lesion)	hypothesis	PET	neuropsychology	non-invasive video-EEG-monitoring	Intracranial EEG	Surgery (reason for deferral)	histopathology	Surgical Outcome (Engel class, duration)
P1	22	f	5	FCD	R F	-	F	R F	-	yes	FCD IIb	I (6 years)
P2	30	f	9	FCD	R I, L O	-	F	no seizures recorded	-	-	-	-
P3	47	m	32	FCD	L F	-	L F-T	L F	L F	yes	FCD IIb	I (min. 2 years)
P4	31	f	3	FCD	L C	L F	L F-T	L F	L F	yes	fragmented tissue, no FCD	currently not classifiable
P5	20	m	19	DNET	R C	R C	L F-T	R F-C	-	- (eloquent area)	-	-
N1	41	m	31	normal	R F	-	no localizing informati on	R F-T	-	-	-	-
N2	28	F	18	normal	L/R T	L T	L F-T	L/R T	L+R T	- (multifocal)	-	-
N3	19	m	10	normal	R T-P-O	normal	R T-P	R T-P-O	R T-P-O	- (eloquent area)	-	-
N4	25	f	21	normal	R F-T	R T	no localizing informati on	R F-T	-	-	-	-
N5	19	m	4	normal	R F-C-T	normal	R F-T	R F-T	-	-	-	-
N6	21	m	14	normal	L T-P	normal	L F	L F-T-P	L T-P	- (eloquent area)	-	-
N7	40	f	1	normal	R I	normal	R F-T	R F	R F/I	- (multifocal)	-	-
N8	20	f	15	normal	R T-P	-	no localizing informati on	R T-P	-	-	-	-
N9	22	f	14	normal	L F	-	L/R F	L F	-	-	-	-
N10	18	f	2	normal	L P-C	-	-	L P-C	-	-	-	-
N11	21	f	12	normal	R F	-	R F-T	R F	-	-	-	-
N12	26	f	3	normal	L T	L T	L F-T	L F-T	L T	yes	no apparent pathology	I D (17 months)

N13	18	f	13	normal	L/R F	normal	L/R F	L/R F	-	-	-	-
N14	27	m	14	normal	L/R T	normal	R F-T	L/R T	-	-	-	-
N15	22	m	16	normal	L/R F	normal	L/R F	L/R F	-	-	-	-
N16	30	f	15	normal	R F-T	-	L/R F	R F-T	-	-	-	-
N17	52	m	45	normal	L F-T	-	-	no localizing informatio n	-	-	-	-
N18	33	m	16	normal	R T	normal	R F-T	R F-T	-	-	-	-
N19	26	m	13	normal	L F	normal	L/R F	L F	-	-	-	-
N20	51	f	33	normal	R T	normal	L F-T	R T	-	- (eloquent area)	-	-
N21	32	f	18	normal	L T	-	L F-T	L T	-	-	-	-
N22	26	w	17	normal	R T	normal	non localizing	R T	-	-	-	-
N23	51	f	30	normal	L/R T	-	L/R F-T	L/R F-T	-	-	-	-
N24	44	m	14	normal	L T	-	L F-T	L/R F-T	-	-	-	-
N25	52	f	35	normal	L T	L/R T	L F-T	L/(R) F-T	-	-	-	-
N26	31	f	19	normal	L T	L T	L/R F	L F-T	L T	yes	no apparent pathology	I A (13 months)
N27	49	m	26	normal	L T	L/R T	L/R F-T	L F-T	L T	- (planned)	-	-

S2 table: Technical MRI sequence parameters

	Tübingen	Göttingen
**scanner**	3T Siemens Magnetom Prisma	3T Siemens Magnetom Prisma fit
**MPRAGE**		
echo time	2.98 ms	3 ms
repetition time	2,300 ms	2,250 ms
inversion time	900 ms	900 ms
flip angle	9°	9°
**MP2RAGE**		
inversion time 1	700 ms	700 ms
flip angle	4°	4°
inversion time 2	2,500 ms	2,500 ms
flip angle	5°	5°
echo time	2.98 ms	2.9 ms
repetition time	5,000 ms	5,000 ms
**T2-SPACE FLAIR**		
echo time	388 ms	394 ms
repetition time	5,000 ms	5,000 ms
inversion time	1800 ms	1800 ms
flip angle	120°	120°

S1 methods: Modeling a random AFROC response

For a large number of subjects, a 'guessing' process that randomly places lesion marks on all subjects can be modeled by a Poisson process, where the expected value of the distribution is equivalent to the FPR (Bandos et al., 2009). Then, the FPF is the complement of the probability of observing a subject with zero false-positive marks (Chakraborty, 2013): $\mathrm{FPF} = 1 - e^{-\mathrm{FPR}}$. When φ is the ratio of ground truth label area to total subject surface area, then $\mathrm{TPF} = 1 - e^{-\varphi \cdot \mathrm{FPR}}$. Replacement of FPR by FPF from the previous equation yields a description of the random response AFROC curve as $\mathrm{TPF} = 1 - (1 - \mathrm{FPF})^{\varphi}$. Integrating results in $\mathrm{AUC} = \int_0^1 \{\mathrm{TPF} \ = \ 1 - (1 - \ \mathrm{FPF})^{\varphi} \,\} d\mathrm{FPF} = \frac{\varphi}{\varphi+1}$.

For smaller numbers of subjects, TPF and FPF for one given threshold (or FPR) can be assumed to follow binomial distributions; however, we are not aware of a way to model the random, 'null' distribution of the entire AFROC curve or of AFROC AUC (Chakraborty, 2013). The chosen Monte Carlo approach also takes into account that in our dataset, φ differs for every subject and that more than one lesion may per present per subject.

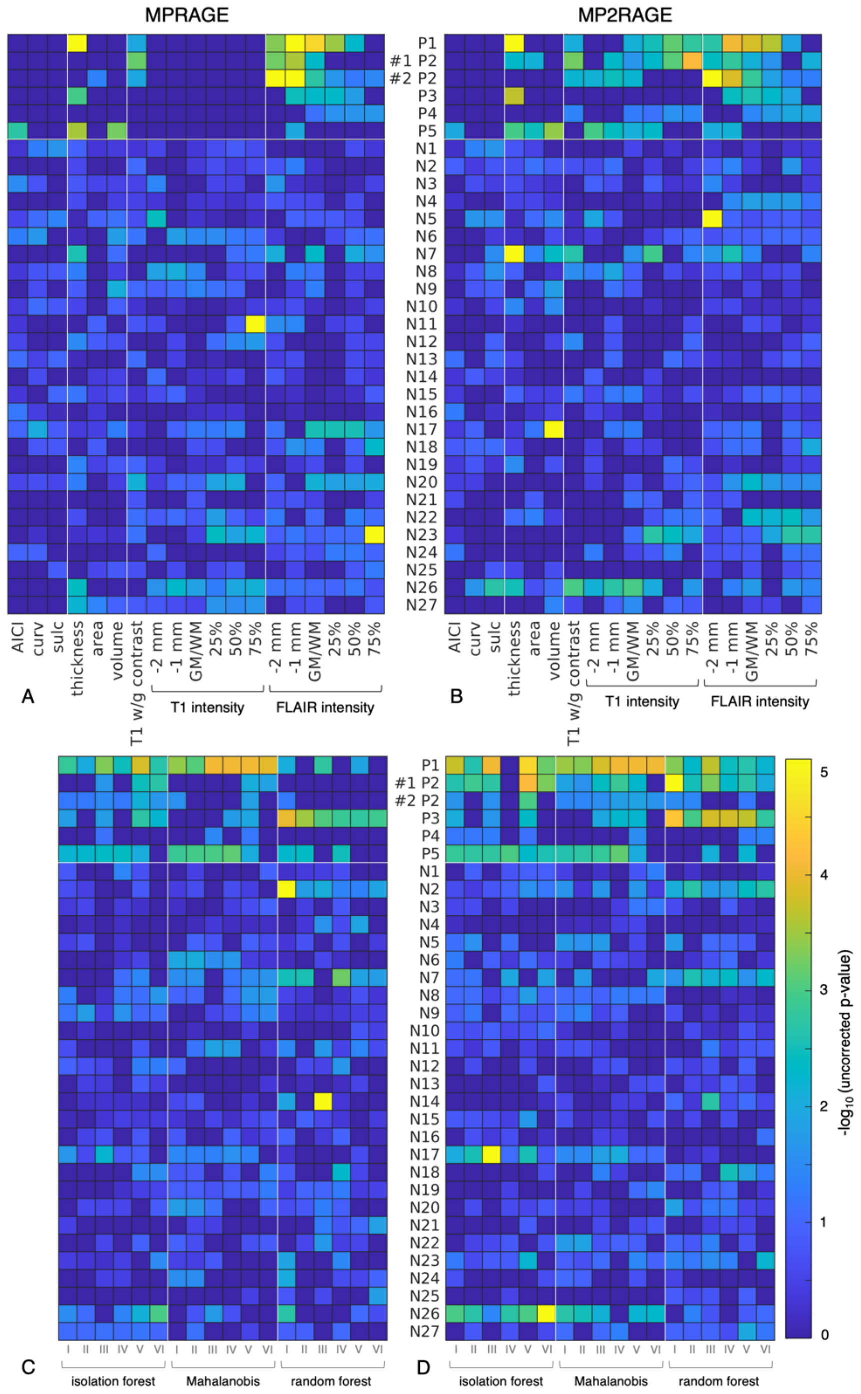

S1 figure: Heatmaps of uncorrected estimated lesion detection p-values for all subjects and detection approaches, (A, B) univariate GLM-based analysis, (C, D) multivariate classifier results where I-VI denotes the input measure subsets as described in figure 6. (A, C) MPRAGE data, (B, D) MP2RAGE. In the MRI-positive patients (subjects P1-P5), it becomes apparent that only some lesions are detected in the thickness maps, whereas FLAIR intensities are useful in others. Normalized T1 intensities are once more only associated with detection performance in MP2RAGE, not in MPRAGE data. In the MRI-negative patients, there are very few instances with very low p-values, likely conveying part of the effect observed on the group level. Detailed data for some of these is explored in figure 8 and supplementary figures 2 and 3.

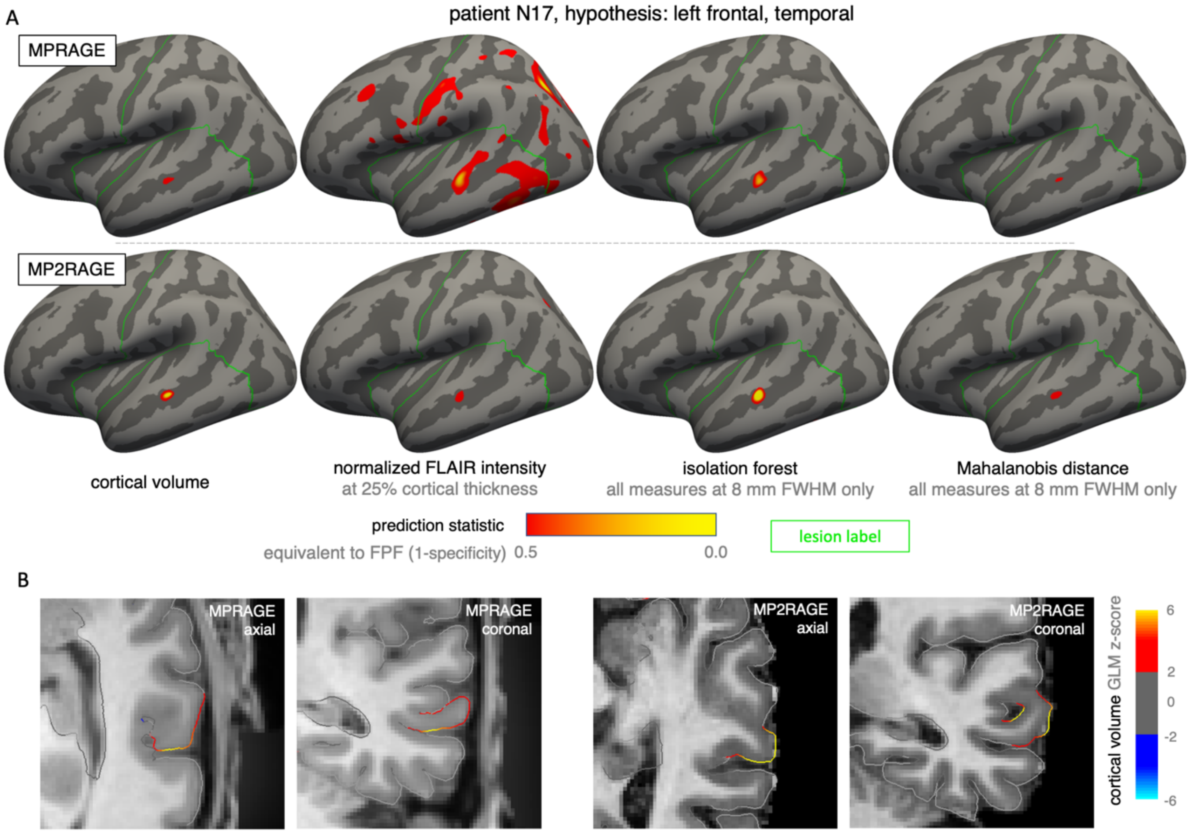

S2 figure: Data from a single MRI-negative subject with low p-values for lesion detection in the MP2RAGE cortical volume, some MPRAGE FLAIR intensity and the MP2RAGE isolation forest III maps. (A) Overlays of the relevant prediction statistic maps on the inflated template surface. (B) Original image data with overlaid tracing of the reconstructed pial surface, colored according to the cortical volume GLM z-score, showing the maximum value in the temporal hypothesis label. Whereas in MPRAGE data, an apparently thick gyrus was highlighted, representing a potentially relevant finding, in MP2RAGE a misclassification of dura caused the finding.

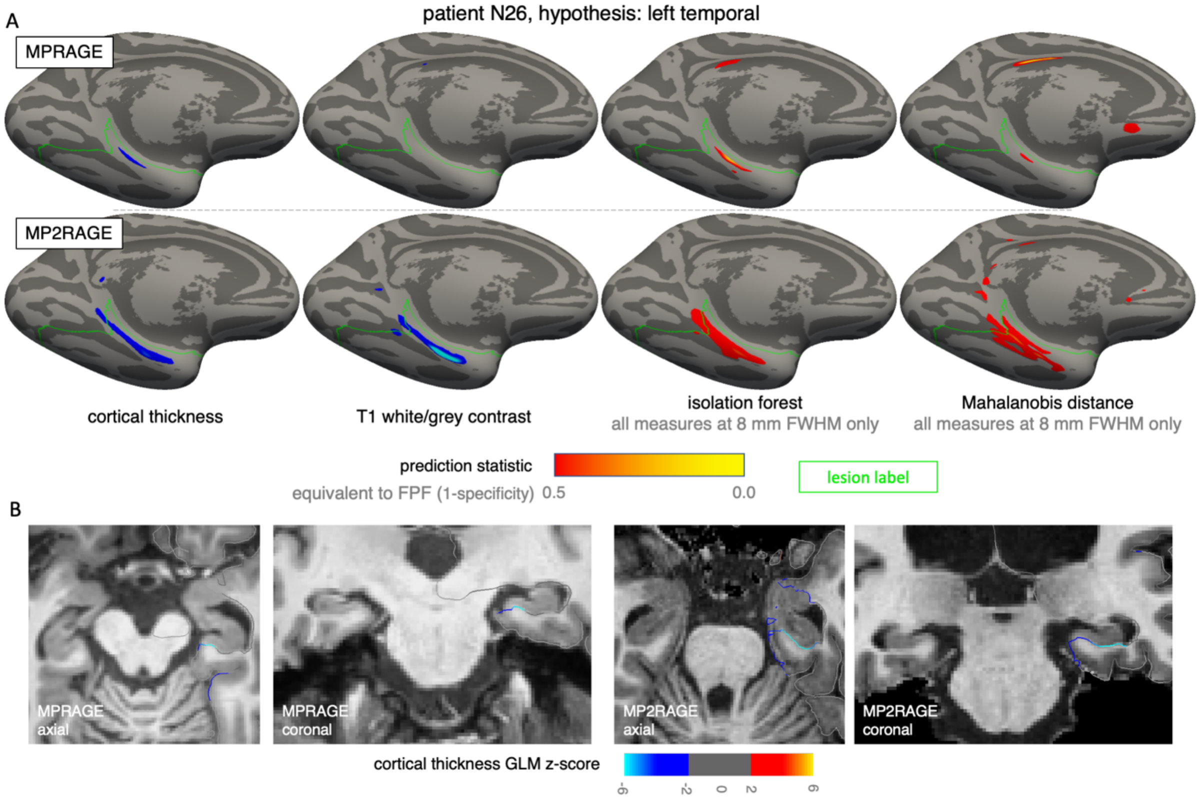

S3 figure: Data from a single MRI-negative subject with low p-values for lesion detection in the cortical thickness, MP2RAGE T1 white-grey contrast and the isolation forest VI maps. (A) Overlays of the relevant prediction statistic maps on the inflated template surface. (B) Original image data with overlaid tracing of the reconstructed pial surface, colored according to the cortical thickness GLM z-score, showing the maximum value in the temporal hypothesis label. The finding localizes to the hippocampus, where Freesurfer thickness estimation is unreliable. This region is meant to be excluded based on the Freesurfer 'cortex' surface label, however this was performed in the template space, coregistration for this subject was apparently inaccurate in this subject, leading to the finding.
